# Mother-to-Child Transmission of Andes Virus through Breast Milk, Chile^1^

**DOI:** 10.3201/eid2608.200204

**Published:** 2020-08

**Authors:** Marcela Ferrés, Constanza Martínez-Valdebenito, Jenniffer Angulo, Carolina Henríquez, Jorge Vera-Otárola, María José Vergara, Javier Pérez, Jorge Fernández, Viviana Sotomayor, María Francisca Valdés, Diego González-Candia, Nicole D. Tischler, Cecilia Vial, Pablo Vial, Gregory Mertz, Nicole Le Corre

**Affiliations:** Pontificia Universidad Católica de Chile, Santiago, Chile (M. Ferrés, C. Martinez-Valdebenito, J. Angulo, C. Henriquez, J. Vera-Otarola, M.J. Vergara, J. Perez, N. Le Corre);; Instituto de Salud Pública de Chile, Santiago (J. Fernandez);; Ministerio de Salud de Chile, Santiago (V. Sotomayor, M.F. Valdes);; Fundación Ciencia & Vida, Santiago (D. Gonzalez-Candia, N. Tischler);; Universidad San Sebastián, Santiago (N. Tischler); Universidad del Desarrollo, Santiago (C. Vial, P. Vial);; University of New Mexico, Albuquerque, New Mexico, USA (G. Mertz)

**Keywords:** Andes virus, hantavirus, viruses, gastrointestinal tract, breast-feeding, transmission, neonatal death, Chile

## Abstract

Andes virus (ANDV) is the only hantavirus transmitted between humans through close contact. We detected the genome and proteins of ANDV in breast milk cells from an infected mother in Chile who transmitted the virus to her child, suggesting gastrointestinal infection through breast milk as a route of ANDV person-to-person transmission.

Andes virus (ANDV), a member of the *Orthohantavirus* genus in the *Hantaviridae* family, has a trisegmented, single-stranded RNA-genome and is the etiologic agent of hantavirus cardiopulmonary syndrome (HCPS) in Chile and Argentina (*1*). The main route of infection in humans is through the inhalation of aerosolized viral particles present in contaminated rodent excreta (*1*), but the virus can also be transmitted from person to person (*2*,*3*). During acute disease, ANDV RNA can be detected in patients’ blood, respiratory secretions, saliva, gingival crevicular fluid, and urine (*2*). Epidemiologic data has suggested that person-to-person transmission mainly occurs through close contact with oral fluids during the prodromal and acute phases of infection (*2*). We report epidemiologic and virologic analyses of a mother in Chile with ANDV infection and apparent transmission to her newborn. The Ethical Review Board of Facultad de Medicina, Pontificia Universidad Católica de Chile, approved the study. 

## The Study

**Figure 1:**
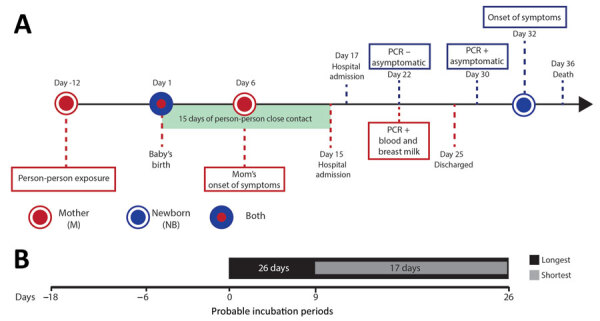
Epidemiologic timeline for mother-to-child transmission of Andes virus through breast milk, Chile. A) Key epidemiologic events related to the mother (represented by M, red circles and lines) and the newborn (NB, blue circle and lines). Blue-and-red circle represents the birth of the newborn; light green rectangle represents the 15 days of close contact that included breastfeeding. We show details for the baby above the time bar and details for the mother below the time bar. B) Longest (black bar) and shortest (light gray bar) probable person-to-person incubation period.

Six days after delivery of a healthy girl, a 21-year-old woman from Parral, Chile, suffered lower-extremity myalgia and weakness. Subsequently, she noted fever of 39.5°C, severe headache, and diaphoresis; she was hospitalized 15 days after delivery. The diagnosis of ANDV infection was confirmed by real-time reverse transcription PCR for ANDV RNA in blood cells, as previously described (*4*). The patient did not report any activities with possible environmental exposure to rodent excreta. However, she had close contact with her father, who had HCPS, while caring for him during his prodromic phase, 12 days before her delivery (Figure 1). She breast-fed and took care of her newborn until day of life (DOL) 15. Although asymptomatic, the newborn was hospitalized for observation at DOL 17; at that time, ANDV IgM testing was negative (Reagenta, https://www.reagena.com). The newborn was screened several times for viral ANDV RNA in blood. On DOL 22, RT-PCR results were negative, but results were positive on DOL 30. Two days later, the baby was transferred to a pediatric intensive care unit with extracorporeal membrane oxygenation availability because of fever. However, severe HCPS developed in the newborn, and she died 4 days later.

Results of ELISA for ANDV-specific IgM/IgG in serum (Euroimmun, https://www.euroimmun.com) were positive for both the mother and the newborn. A breast milk sample tested positive for ANDV RNA on day 16 after the mother’s first symptoms; previous samples of breast milk were not available. We also tested other body fluids from the newborn, including urine, saliva, and cerebrospinal fluid for ANDV RNA by real-time RT-PCR (Appendix Table).

ANDV RNA has previously been detected in bodily fluids other than blood, such as saliva, respiratory secretions, and urine (*2*). Therefore, close contact with such fluids may explain additional cases for which high-risk environmental and rodent exposure is absent or improbable. In our study of the infected newborn, we ruled out environmental exposure; the only possible source was close contact to her mother during the incubation period and initial clinical disease. The mother maintained breast-feeding until the baby was hospitalized and confirmed to be viremic.

**Figure 2:**
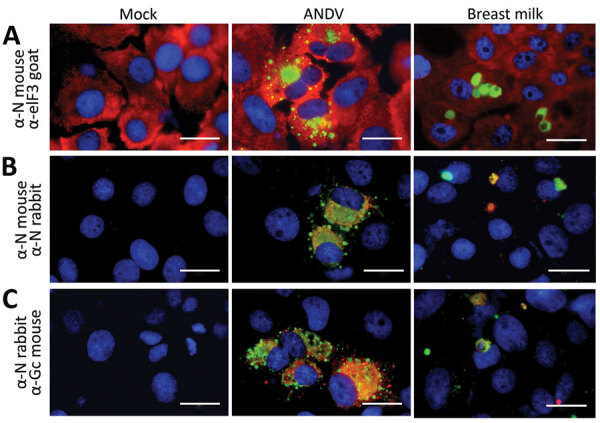
Detection of ANDV N and Gc proteins from enucleated cells from breast milk from a mother in Chile. A) Detection of N protein and the cytoplasmic marker eIF3. B) Detection of N protein with 2 different primary antibodies. C) Detection of N and Gc proteins. Huh-7 cells were mock-infected (mock column), ANDV infected (ANDV column), or incubated with a pellet from breast milk (breast milk column). Coverslips were incubated with mouse and rabbit antibodies. Scale bars indicate 20 µm. Complete methods are described in the Appendix. ANDV, Andes virus; Gc, glycoprotein; N, nucleoprotein.

To evaluate the presence of ANDV particles in breast milk, we performed a culture in Huh-7 cells mock-infected and incubated with a breast milk pellet and, as a positive control, ANDV at a multiplicity of infection of 1 (*5*). After infection, we identified viral nucleoprotein (N) and glycoprotein (Gc) through immunofluorescence assay (Appendix). We detected N protein in the cytoplasmic compartment of ANDV-infected cells and cells incubated with breast milk but not in mock-infected cells (Figure 2, panel A). To verify detection specificity, we used 2 different ANDV N protein antibodies generated in mice and rabbits. Again, we identified N protein in ANDV-infected cells and incubated with breast milk but not in mock-infected cells (Figure 2, panel B). Moreover, N and Gc proteins were only detectable in ANDV-infected cells and cells incubated with breast milk (Figure 2, panel C). Of interest, we did not stain ANDV-infected cells from breast milk by 4′,6-diamidino-2- phenylindole (DAPI, Vectashield H1200; Vector Laboratories, Inc, https://vectorlabs.com) (Appendix Figures 1, 2); the nuclear compartment of mock-infected Huh-7 cells and ANDV positive control were stained by DAPI. ANDV-infected cells from breast milk (8 µm [SD +1.2 µm] in diameter on the basis of 10 cells from different captured fields) were clearly smaller in size than the Huh-7 cells (27 µm [SD + 4.3 µm]). Altogether, our results demonstrate the presence of ANDV in enucleated breast milk cells.

Breast milk contains a variety of blood cells (monocytes, T-cells, NK cells, B cells, and neutrophils) and hematopoietic stem cells (*6*). In this context, we know that ANDV can be present in buffy coat cells for up to 15 days before illness onset (*7*); is always present during the acute phase of the disease, including the febrile prodrome phase; and remains in a small proportion of cases during convalescence (*7*). Assuming that breast milk contained ANDV-infected cells, direct inoculation in Peyer’s patches in the newborn may have resulted in virus entry (*8*). Another possible mode of transmission is close contact with respiratory secretions and saliva of the infected mother. However, because ANDV was present in breast milk and the newborn’s exposure to breast milk was much greater than to other fluids, transmission by breast milk is very likely.

Another factor that may help explain oral infection in the neonatal period is the gastrointestinal characteristics in the first month of life, such as the adjustment of stomach pH, rapid gastric emptying time, and increased permeability of the intestine due to loosened intestinal intercellular spaces (*9*). Sin Nombre virus was detected in breast milk samples by RT-PCR, but the exposed child did not become infected (*10*). Similar results were found in 2 women infected with Puumala virus (*11*). Vertical transmission was excluded in 4 pregnant women infected with hantavirus species in Europe (*12*). Bellomo et al. reported a newborn infected with ANDV but did not report the presence of ANDV in breast milk (*13*). Our case provides further evidence for a gastrointestinal transmission of ANDV, which is consistent with previous reports of Puumala virus and ANDV infections in Syrian hamster models of hantavirus cardiopulmonary disease and in 1 newborn human case (*13*–*15*).

## Conclusions

We describe mother-to-child transmission of ANDV infection in Chile. Our analyses proved the presence of ANDV in breast milk, proposing breast-feeding as an additional mechanism of transmission. In this context, we recommend that ANDV-infected mothers refrain from breast-feeding until ANDV RNA is undetectable in blood and breast milk. In addition, we advise strict clinical and virologic surveillance of children potentially exposed to family members with ANDV infection for early diagnosis and hospitalization for adequate intensive care.

Appendix Additional information for mother-to-child transmission of Andes virus through breast milk, Chile.
