# Supplementary material for: Mother-to-Child Transmission of Andes Virus through Breast Milk, Chile
Source: Emerg Infect Dis. 2020 Aug;26(8):1885–8. doi: 10.3201/eid2608.200204 (PMC7392419; doi:10.3201/eid2608.200204)
Supplement: Appendix — Additional information for mother-to-child transmission of Andes virus through breast milk, Chile. [file 20-0204-Techapp-s1.pdf]

Article DOI: https://doi.org/10.3201/eid2608.200204

Mother-to-Child Transmission of Andes Virus through Breast Milk, Chile

Appendix

Materials and Methods

Patients

Mother and newborn were enrolled under 3 research protocols (funded by Comision nacional de investigacion cientifica y tecnológica, Fondecyt, nos. 1161197 and 1161447). Clinical and epidemiologic variables were obtained from clinical charts and personal interviews with the mother and grandmother using ad hoc questionnaires. The mother signed a consent form to use their clinical, laboratory and epidemiologic data for this study. The study was approved by the Ethical Review Board, Facultad de Medicina, Pontificia Universidad Católica de Chile.

Sample Collection and Prospective Follow-Up of Asymptomatic Newborn

We collected peripheral blood at hospital admission from the mother, as well as saliva, cerebrospinal fluid, stool, and urine samples from the newborn. Also, 25 mL of breast milk were collected 16 days after symptom onset and stored at −80°C.

A weekly follow-up examination of the baby was planned for 6 weeks, starting the day the mother was hospitalized, with the goal of early detection of ANDV RNA in blood.

Nucleic Acid Purification

Nucleic acids for ANDV viral detection in peripheral blood (buffy coat), peripheral mononuclear cells (PBMCs), saliva, cerebrospinal fluid, stool, urine and breast milk were extracted using MagNA Pure System (Roche, https://www.roche.com) according to the manufacturer’s instructions; samples were stored at −20°C.

The breast milk was centrifuged and the supernatant and pellet were aliquoted and stored at −80°C. Huh-7 cells, kindly provided by Dr. R. Bartenschlager, University of Heidelberg, in a 24-well format were mock- infected or incubated with 80 µL of breast milk pellet (ratio 1:1 with

media, 160 μL final volume) for 1.5 hours; then, 2 PBS washes were performed and fresh medium was added. As a positive control, cells were infected with ANDV for 1 hour at a multiplicity of infection of 1. All the procedures were performed under Biosafety Level 3 (BSL-3) conditions.

Indirect immunofluorescence (IF)

Cells were fixed with paraformaldehyde (PFA; Merck, https://www.merck.com) 4% in PBS for 10 min, permeabilized with PBS- Triton 0.03% for 10 min, and blocked with BSA 10% in PBS for 1 hour, all at room temperature (RT). Coverslips were incubated with combinations of antibodies, such as ANDV N mouse monoclonal antibodies (clone 7B3/F7) (*1–3*), ANDV N rabbit polyclonal antibodies (*3*), ANDV Gc monoclonal antibodies (clone 2H4/F6) (*1*), and eIF3n goat polyclonal antibodies (sc-16377; Santa Cruz Biotechnology, https://www.scbt.com), in BSA 5% and PBS-Triton 0.03% overnight at 4°C. For final detection, we used donkey anti-mouse Alexa-488 (green), donkey anti-goat Alexa 594 (red) and donkey anti-rabbit Alexa 594 as secondary antibodies (Invitrogen, https://www.thermofisher.com) for 1 hour at RT. Vectashield H1200 (Vector Laboratories, Inc, https://vectorlabs.com) with DAPI (blue) was used as mounting media, and samples were sealed and stored at 4°C. An Olympus model BX51 microscope was used to capture images using a 60x objective with an MBF Stereo Investigator version 11.

References

1. Godoy P, Marsac D, Stefas E, Ferrer P, Tischler ND, Pino K, et al. Andes virus antigens are shed in urine of patients with acute hantavirus cardiopulmonary syndrome. J Virol. 2009;83:5046–55. PubMed https://doi.org/10.1128/JVI.02409-08

2. Tischler ND, Rosemblatt M, Valenzuela PD. Characterization of cross-reactive and serotype-specific epitopes on the nucleocapsid proteins of hantaviruses. Virus Res. 2008;135:1–9. PubMed https://doi.org/10.1016/j.virusres.2008.01.013

3. Vera-Otarola J, Solis L, Soto-Rifo R, Ricci EP, Pino K, Tischler ND, et al. The Andes hantavirus NSs protein is expressed from the viral small mRNA by a leaky scanning mechanism. J Virol. 2012;86:2176–87. PubMed https://doi.org/10.1128/JVI.06223-11

Appendix Table. Virologic and serologic analysis of fluids from mother and newborn during acute stage of hantavirus cardiopulmonary syndrome*

	ANDV RT-PCR result (days past onset of symptoms						IgM/IgG ANDV	
Case	Blood	Breast milk	Saliva	Urine	CSF	Stool	Blood	CSF
Mother	Pos (+9 to +29)	Pos (+16 and+24)	Neg (+29)	NA	NA	NA	Pos/NA (+9)	NA
Newborn	Pos (-2 to +3)	NA	Neg (+3)	Pos (+3)	Neg (+3)	Neg	Pos/Pos (+3)	Pos/Pos (+3)

*ANDV, Andes virus; CSF, cerebrospinal fluid; NA, not available; Neg, negative; Pos, positive; RT-PCR, reverse transcription PCR.

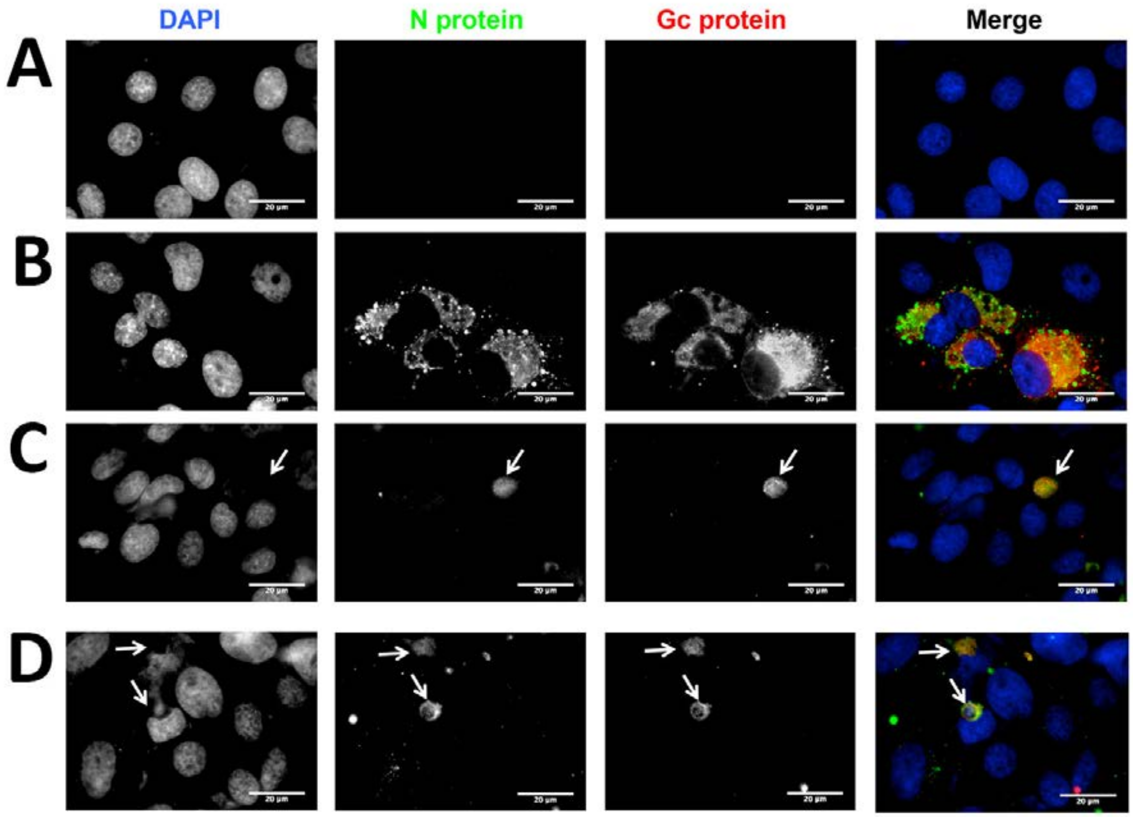

Appendix Figure 1. Cells in breast milk harbor Andes virus (ANDV) N and Gc proteins. Huh-7 cells were mock-infected (A) or infected with ANDV (B). In rows C and D, the Huh-7 cells were incubated for 1 hour with a pellet of cells from breast milk. Twenty-four hours later, the cells were fixed with PFA 4% and permeabilized with PBS-Triton 0.003%. Coverslips were incubated with rabbit polyclonal N antibodies and mouse monoclonal Gc antibodies. Alexa anti rabbit-488 and Alexa anti mouse-594 were used as secondary antibodies. Vectashield (Vector Laboratories, Inc, https://vectorlabs.com) with DAPI was used as mounting media. The images were obtained with an Olympus epifluorescence microscope and processed by ImageJ software (U S National Institutes of Health, , https://imagej.nih.gov/ij). The white arrows show the ANDV-infected breast milk cells in different channels. For all panels in the figure, size bars correspond to 20 µm.

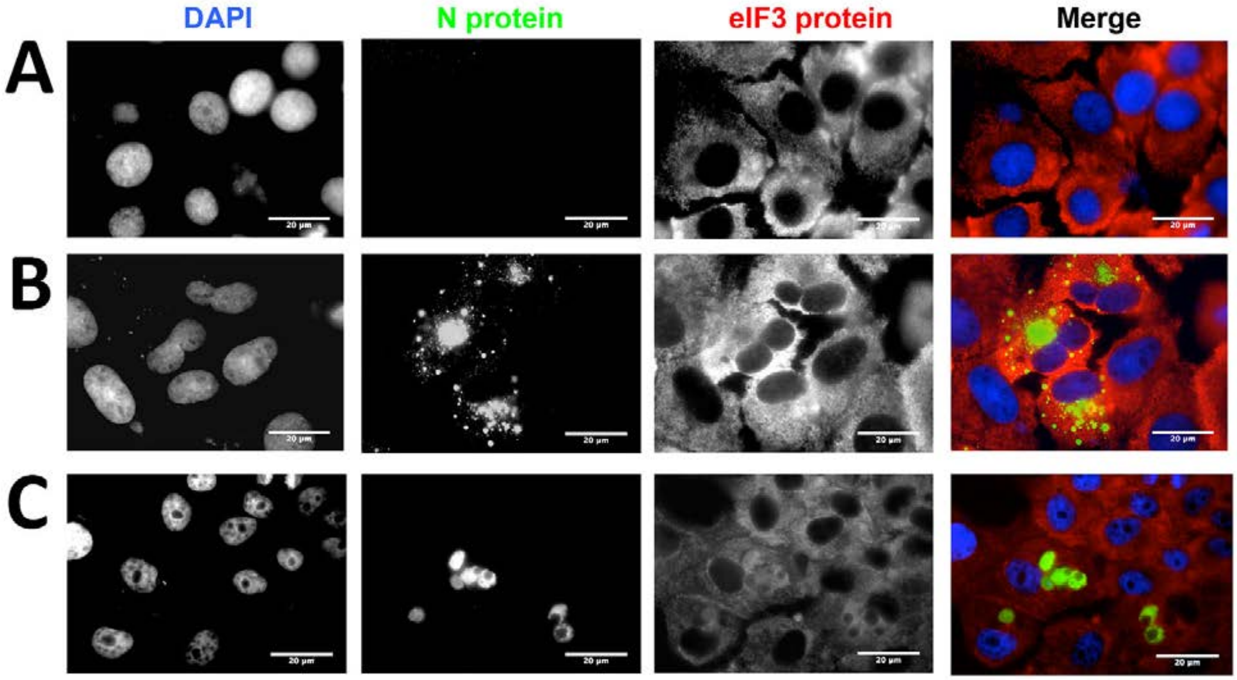

Appendix Figure 2. Enucleated cells from breast milk harbor Andes virus (ANDV) N protein. Huh-7 cells were mock-infected (A) or infected with ANDV (B). In row C, the Huh-7 cells were incubated for 1 hour with a pellet of cells from breast milk. Twenty-four hours later, the cells were fixed with PFA 4% and permeabilized with PBS-Triton 0.003%. Coverslips were incubated with mouse monoclonal N antibodies and goat polyclonal eIF3 antibodies. Anti-mouse 488 and anti-goat 594 were used as secondary antibodies. Vectashield with DAPI was used as mounting media. The images were obtained with an Olympus epifluorescence microscope and processed by ImageJ software. For all panels in the figure, size bars correspond to 20 µm.
